# Sequence analysis of two F1 mycobacteriophages, Deb65 and DocMcStuffins

**DOI:** 10.1128/mra.01328-24

**Published:** 2025-04-14

**Authors:** Marcus O. Royster, Victoria Figgins, Vera Pande, Jason D. Robinson, Deeka S. Abdi, Ali Amin, Zephaniah Ansah, Ethan W. Bomersheim, Gianna Dunn, Ali A. Elfaki, Jordyn Foulk, Kate C. Ingle, Avi D. Lavu, Ved Pande, Priya T. Shan, Marie P. Smithbey, Gunnar R. Ternstrom, Olivia S. Trager, David A. Washington, Monica Xu, Margaret S. Saha

**Affiliations:** 1 Department of Biology, William and Mary 402696, Williamsburg, Virginia, USA; Department of Biology, Queens College, New York, USA

**Keywords:** *Mycobacterium smegmatis*, mycobacteriophage

## Abstract

Isolated from wetland soil, Deb65 and DocMcStuffins are bacteriophages with a siphoviral morphology that infect *Mycobacterium smegmatis*. Deb65 and DocMcStuffins encode 97 and 91 putative genes, 41 of which are shared. Based on gene content similarity to actinobacteriophages more broadly, both phages are assigned to subcluster F1.

## ANNOUNCEMENT

Advancing our knowledge of mycobacteriophage diversity is essential for understanding the abundance, community dynamics, and evolution of Mycobacteria, an environmentally and clinically important genus (1–5). We report the sequence of two genetically distinct mycobacteriophages, Deb65 and DocMcStuffins.

**Fig 1:**
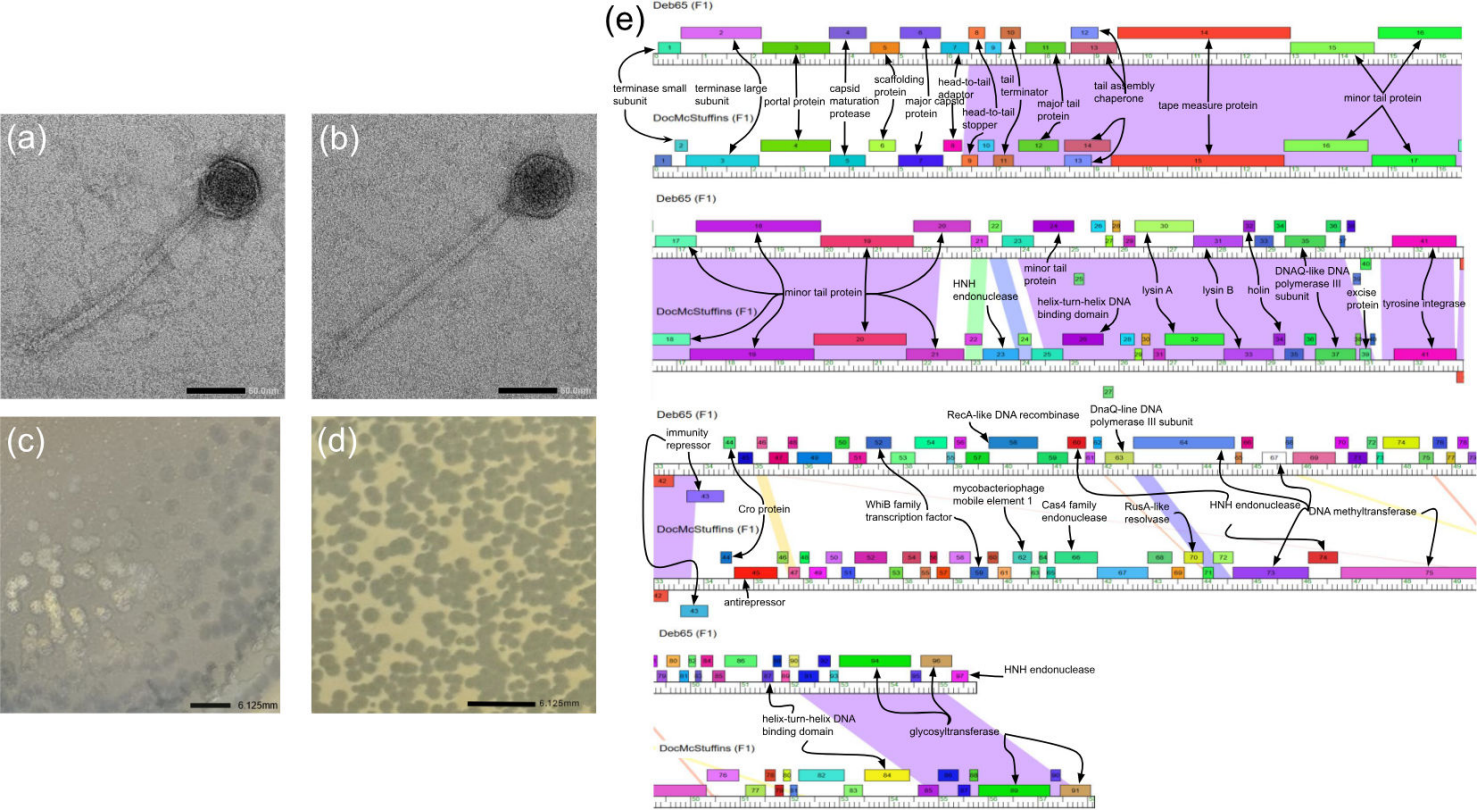
(a) Negative stain (uranyl acetate, 1%) transmission electron microscopy of DocMcStuffins revealing siphoviral morphology. Scale bar is 50 nm. (b) Negative stain (uranyl acetate, 1%) transmission electron microscopy of Deb65 revealing siphoviral morphology. Scale bar is 50 nm. (c) Plaque image of DocMcStuffins showing clear plaques. Scale bar is 6.125 mm. (d) Plaque image of Deb65 showing clear plaques. Scale bar is 6.125 mm. (e) Phamerator comparative alignment of Deb65 and DocMcStuffins.

Both phages were isolated from wet, silty soil samples at the College of William & Mary in Williamsburg, VA, USA, using a standard enrichment procedure (Table 1) (6). Briefly, 5 g of each soil sample was suspended in 50 mL of 7H9 media, inoculated with *Mycobacterium smegmatis* mc^2^ 155, and incubated in a 37°C shaker (250 rpm) for 2 days. The resulting cultures were filtered through a 0.22 µm filter, and the filtrates were plated with *M. smegmatis* in 7H9 top agar, yielding clear plaques for both phages, Deb65 and DocMcStuffins, after 24–48 h. Phages were then purified through three rounds of plating before being imaged by negative stain (1% uranyl acetate) transmission electron microscopy to reveal siphoviral morphologies for both phages (Fig. 1).

**TABLE 1:** Genome and sequencing information for DocMcStuffins and Deb65

Phage	DocMcStuffins	Deb65
Isolation GPS	37°16′08.4″N 76°43′08.4″W	37°16′15.1″N 76°42′56.7″W
Morphology	Siphovirus	Siphovirus
Capsid diameter	50 ± 3 nm (*n* = 5)	50 ± 2 nm (*n* = 5)
Tail length	~180 ± 5 nm (*n* = 7)	~190 ± 5 nm (*n* = 5)
Sequencing reads	453,180	440,517
Sequencing coverage, fold	1,122	1,119
Genome length (bp)	58,159	55,767
Genome end sequence	5′ CCGAAGGCAT	5′ CGGACGGCGC
Number of open reading frames	91	97
GC content (%)	62.7	61.6
Accession number	PQ184804	PQ184836
SRA	SRX26785850	SRX26785849

Phage DNA was extracted from a lysate using the phenol-chloroform-isoamyl alcohol method and ethanol precipitated (7). DNA was prepared for sequencing using the NEB Ultra II Library Kit and sequenced using an Illumina MiSeq Sequencer (v3 reagents, single-end, 150 base read). Newbler (version 2.9) was then used to assemble the genome and Consed (version 29) to check for completeness and reveal 3′ single-stranded genome termini (8). Sequencing data, overhangs, and genome characteristics are presented in Table 1.

The genomes were annotated using DNA Master (version 5.23.6) and PECAAN (version 20221109) (9). Translational start sites were verified using the coding potential predicted by GeneMark and Glimmer (10–12), and the similarity of start sites in homologs was identified using Starterator (http://phages.wustl.edu/starterator/) and BLASTp against the Actinobacteriophage and NCBI non-redundant protein databases (6, 13). No tRNAs were identified using Aragorn version 1.2.41 (14) and tRNAscan (15).

Putative gene functions were assigned based on predictions from HHPred (using the PDB_mmCIF70, NCBI_CD, SCOPe70, and pFAM-A as databases), BLASTp, and Phamerator (Actino_draft database) for highly similar genes (16, 17). Using the gene content similarity (GCS) tool at the Actinobacteriophage database, phagesDB (https://phagesdb.org/) and clustering parameters of at least 35% GCS to actinobacteriophages, both phages are assigned to cluster F, subcluster F1 (18, 19). Default settings were used for all software.

Both phages share 41 GCS, which are primarily in the first half of the genome and include genes encoding functions in virion structure, assembly, lysis, and lysogeny, the latter consistent with the temperate lifecycle for F cluster phages (Fig. 1). Noteworthy here is that while both phages encode homologous tyrosine integrases with 90% amino acid identity (AAI), their immunity repressors only share 39% AAI. Within this genomic region, and consistent with many phages of the F1 subcluster, both Deb65 and DocMcStuffins encode anti-repressor and Cro proteins. Within the second half of the genome, where gene conservation is lower, both phages encode two glycosyltransferases that are highly conserved across the F1 subcluster. Within this region, Deb65 encodes a unique DNA methyltransferase for which no homolog exists in the Actinobacteriophage database.

## Data Availability

Deb65 and DocMcStuffins are available at GenBank with accession nos. PQ184836 and PQ184804, and Sequence Read Archive (SRA) nos. SRX26785849 and SRX26785850.
